# Cytokine Profiles Associated With Acute COVID-19 and Long COVID-19 Syndrome

**DOI:** 10.3389/fcimb.2022.922422

**Published:** 2022-06-30

**Authors:** Maria Alice Freitas Queiroz, Pablo Fabiano Moura das Neves, Sandra Souza Lima, Jeferson da Costa Lopes, Maria Karoliny da Silva Torres, Izaura Maria Vieira Cayres Vallinoto, Carlos David Araújo Bichara, Erika Ferreira dos Santos, Mioni Thieli Figueiredo Magalhães de Brito, Andréa Luciana Soares da Silva, Mauro de Meira Leite, Flávia Póvoa da Costa, Maria de Nazaré do Socorro de Almeida Viana, Fabíola Brasil Barbosa Rodrigues, Kevin Matheus Lima de Sarges, Marcos Henrique Damasceno Cantanhede, Rosilene da Silva, Clea Nazaré Carneiro Bichara, Ana Virgínia Soares van den Berg, Adriana de Oliveira Lameira Veríssimo, Mayara da Silva Carvalho, Daniele Freitas Henriques, Carla Pinheiro dos Santos, Juliana Abreu Lima Nunes, Iran Barros Costa, Giselle Maria Rachid Viana, Francisca Regina Oliveira Carneiro, Vera Regina da Cunha Menezes Palacios, Juarez Antonio Simões Quaresma, Igor Brasil-Costa, Eduardo José Melo dos Santos, Luiz Fábio Magno Falcão, Antonio Carlos Rosário Vallinoto

**Affiliations:** ^1^ Laboratório de Virologia, Instituto de Ciências Biológicas, Universidade Federal do Pará, Belém, Brazil; ^2^ Instituto de Ciências Biológicas e da Saúde, Universidade do Estado do Pará, Belém, Brazil; ^3^ Programa de Pós-Graduação em Biologia de Agentes Infecciosos e Parasitários, Universidade Federal do Pará, Belém, Brazil; ^4^ Laboratório de Genética de Doenças Complexas, Instituto de Ciências Biológicas, Universidade Federal do Pará, Belém, Brazil; ^5^ Hospital Adventista de Belém, Belém, Brazil; ^6^ Seção de Arbovirologia e Febres Hemorrágicas, Instituto Evandro Chagas, Secretária de Vigilância em Saúde, Ministério da Saúde do Brasil, Ananindeua, Brazil; ^7^ Laboratório de Imunologia, Seção de Virologia, Instituto Evandro Chagas, Secretária de Vigilância em Saúde, Ministério da Saúde do Brasil, Ananindeua, Brazil; ^8^ Laboratório de Pesquisas Básicas em Malária em Malária, Seção de Parasitologia, Instituto Evandro Chagas, Secretária de Vigilância em Saúde, Ministério da Saúde do Brasil, Ananindeua, Brazil

**Keywords:** SARS-CoV-2, COVID-19, long COVID-19, risk factor, cytokines

## Abstract

The duration and severity of COVID-19 are related to age, comorbidities, and cytokine synthesis. This study evaluated the impact of these factors on patients with clinical presentations of COVID-19 in a Brazilian cohort. A total of 317 patients diagnosed with COVID-19 were included; cases were distributed according to clinical status as severe (n=91), moderate (n=56) and mild (n=170). Of these patients, 92 had acute COVID-19 at sample collection, 90 had already recovered from COVID-19 without sequelae, and 135 had sequelae (long COVID syndrome). In the acute COVID-19 group, patients with the severe form had higher IL-6 levels (p=0.0260). In the post-COVID-19 group, there was no significant difference in cytokine levels between groups with different clinical conditions. In the acute COVID-19 group, younger patients had higher levels of TNF-α, and patients without comorbidities had higher levels of TNF-α, IL-4 and IL-2 (p<0.05). In contrast, patients over age 60 with comorbidities had higher levels of IL-6. In the post-COVID-19 group, subjects with long COVID-19 had higher levels of IL-17 and IL-2 (p<0.05), and subjects without sequelae had higher levels of IL-10, IL-6 and IL- 4 (p<0.05). Our results suggest that advanced age, comorbidities and elevated serum IL-6 levels are associated with severe COVID-19 and are good markers to differentiate severe from mild cases. Furthermore, high serum levels of IL-17 and IL-2 and low levels of IL-4 and IL-10 appear to constitute a cytokine profile of long COVID-19, and these markers are potential targets for COVID-19 treatment and prevention strategies.

## Introduction

Coronavirus disease 2019 (COVID-19) has had a great impact on people’s lives worldwide. The disease and its consequences have been the cause of death for more than 5 million individuals in the past two years (WHO, 2022). Infection caused by severe acute respiratory syndrome coronavirus 2 (SARS-CoV-2) is responsible for COVID-19, which is characterized by symptoms ranging from fever to the development of severe acute respiratory syndrome (SARS) (Wang et al., 2020). The presence of different signs and symptoms determines the different clinical conditions of the disease, which can be asymptomatic, mild, moderate, severe or critical (WHO, 2021).

In most patients, SARS-CoV-2 infection is asymptomatic, especially in children (approximately 80%), but 20% of patients require admission to the intensive care unit (ICU); the mortality rate of these patients is 25%, with most deaths attributed to severe inflammation and thromboembolic complications (Buszko et al., 2020).

The duration and severity of COVID-19 is related to several factors, including viral (mutations) (Nagy et al., 2021) and host (age, sex, comorbidities and immunological) factors (Wang et al., 2021; Chang et al., 2021). SARS-CoV-2 infection begins with its binding to the ACE2 protein of alveolar epithelial cells, which induces activation of the innate and adaptive immune responses through the production and interaction of chemokines, colony-stimulating factors, interferons, interleukins, and tumor necrosis factor-α (TNF-α). These factors increase vascular permeability, determining COVID-19 development (Kaur et al., 2021; Torres et al., 2022).

As immunity is the essential component that determines the type of interaction between the pathogen and the host in any infectious disease; when the immune response is dysregulated, it contributes to disease pathogenesis, as in the case of a “cytokine storm” (Chen et al., 2021).

The term “cytokine storm” was coined to describe intense production of cytokines in infectious processes responsible for triggering immunopathological reactions (Teijaro, 2017). Among the inflammatory mediators released by immune cells, the cytokines IFN-α, IFN-γ, IL-1β, IL-6, IL-12, IL-18, IL-33, TNF-α and TGF-β are highlighted, and altered levels are associated with different clinical features of COVID-19 (Huang et al., 2021a; Chang et al., 2021). Indeed, cytokine storms correlate with the severity and progression of COVID-19 and can result in serious complications such as acute respiratory distress syndrome (ARDS) and multiple organ failure, which are the leading causes of death from the disease (Kunnumakkara et al., 2021).

Another complexity of COVID-19 is the emergence of new symptoms after the acute infection or illness. Patients infected with SARS-CoV-2 may continue to experience a variety of symptoms after the established period of COVID-19, which are not explained by other causes. These symptoms include fatigue, shortness of breath, “brain fog”, sleep disturbances, fevers, gastrointestinal symptoms, anxiety and depression and can persist for months and range from mild to disabling (Collins, 2021; Parums, 2021).

The immune response is a critical factor in the evolution of COVID-19, and assessment of this response in different populations can provide a better understanding of how the host response influences the severity of the disease in some individuals, even though the majority of those infected with SARS-CoV-2 are asymptomatic or develop mild symptoms (Mortaz et al., 2020). Accordingly, we evaluated the levels of IL-17, IFN-γ, TNF-α, IL-10, IL-6, IL-4 and IL-2 in patients with clinical COVID-19 and long COVID-19, with cases classified as mild, moderate and severe, and associations with risk factors for sex, age and presence of comorbidities.

## Materials and Methods

### Study Population and Sample Collection

The present study involved blood samples from 317 patients diagnosed with COVID-19 and classified according to the criteria established by the World Health Organization (WHO, 2021). The assessment included individuals of both sexes aged 18 years and over, who had not been vaccinated against SARS-CoV-2, and who were attended at the post-COVID-19 outpatient clinic at Universidade do Estado do Pará, Hospital Adventista de Belém or Instituto Evandro Chagas from July 2020 to May 2021. The group of post-COVID-19 patients included those who sought the long COVID-19 outpatient clinic at least 30 days after the recovery from acute COVID-19.

A total of 317 patients (n=317) were enrolled in our study aiming to access the cytokine profiles according to the symptoms severe (n=91), moderate (n=53) and mild (n=173), presented in the moment of acute disease. Aiming to analyze the cytokine profile among recovered patients of COVID-19 (n=225) that looked attending the long COVID-19 outpatient clinic at the Universidade do Estado do Pará, we divided the individuals into two groups: post-COVID-19 with (n=135) and without (n=90) sequelae (long COVID-19).

A 10-mL blood sample was collected by intravenous puncture using a vacuum collection system containing ethylenediaminetetraacetic acid (EDTA) as an anticoagulant. The samples were transported to the Virology Laboratory of the Federal University of Pará, where they were processed to separate plasma and leukocytes; the former was used to determine cytokine levels.

### Serum Cytokine Levels

Quantification of serum cytokine levels was performed using the flow cytometry technique and Cytometric Bead Array Kit (CBA) Human Th1/Th2/Th17 (BD Biosciences, San Diego, CA, USA) with BD FACS Canto II equipment. All procedures followed the manufacturer’s guidelines. The methodology used is based on beads conjugated with a capture antibody: six populations of beads with different fluorescence intensities conjugated to a specific capture antibody for each cytokine are mixed to form the CBA, and results are determined using the FL-3 channel of a flow cytometer. The bead populations were observed according to their respective fluorescence intensities: from least bright to brightest (IL-17< IFN-γ < TNF-α < IL-10 < IL-6 < IL-4 < IL-2).

### Statistical Analysis

The information obtained was entered into a database in Microsoft Office Excel 2013 software. Normality of the distribution of cytokine levels was analyzed using the Shapiro–Wilk test. Based on the results of the normality test, the evaluation of variations in the plasma levels of these markers between groups was performed using the nonparametric Mann–Whitney test. RStudio 4.0.2 software was used to assess the correlation (Pearson correlation) between IL-6 levels and age in the acute COVID-19 group. The frequency of epidemiological variables was estimated using direct counting, and the significance of differences between the groups was calculated using Fisher’s exact test and the chi-square test. All tests were performed using the GraphPad Prism 5.0 program, and results with a p value < 0.05 were considered significant.

## Results

**Table 1** shows the median cytokine levels of patients with severe and mild/moderate acute COVID-19 (acute SARS-CoV-2 infection) and individuals who recovered from the disease (with and without sequelae).

**Table 1:** - Median cytokine levels evaluated among patients with different clinical COVID-19 and post-COVID-19 conditions.

Clinical condition	IL-17Median (IQR)	IFN-γMedian (IQR)	TNF-αMedian (IQR)	IL-10Median (IQR)	IL-6Median (IQR)	IL-4Median (IQR)	IL-2Median (IQR)
**COVID-19**	15.45 (15.90)	8.13 (3.15)	6.20 (3.25)	9.50 (3.21)	12.22 (8.31)	8.83 (15.90)	6.88 (7.57)
**Post-COVID-19**	20.46 (15.59)	8.54 (2.51)	7.92 (4.65)	9.64 (3.19)	8.58 (4.47)	8.73 (89.48)	8.44 (2.72)
**COVID-19**							
Severe	15.80 (16.24)	8.42 (2.94)	6.17 (3.66)	9.46 (3.22)	13.53 (11.35)	8.04 (5.72)	6.81 (2.46)
Mild/Moderate	15.16 (15.28)	7.32 (3.58)	6.30 (3.19)	9.50 (4.14)	10.98 (5.01)	9.31 (5.72)	8.18 (2.60)
**Post-COVID-19**							
Severe	20.41 (13.09)	8.34 (3.76)	6.85 (6.76)	8.78 (2.95)	8.22 (4.6)	7.59 (7.61)	7.60 (3.26)
Mild/Moderate	20.50 (15.85)	8.57 (2.26)	8.23 (4.2)	9.75 (3.24)	8.67 (4.47)	8.83 (7.89)	8.60 (2.66)

IIQ, interquartile range.

**Figure 1:**
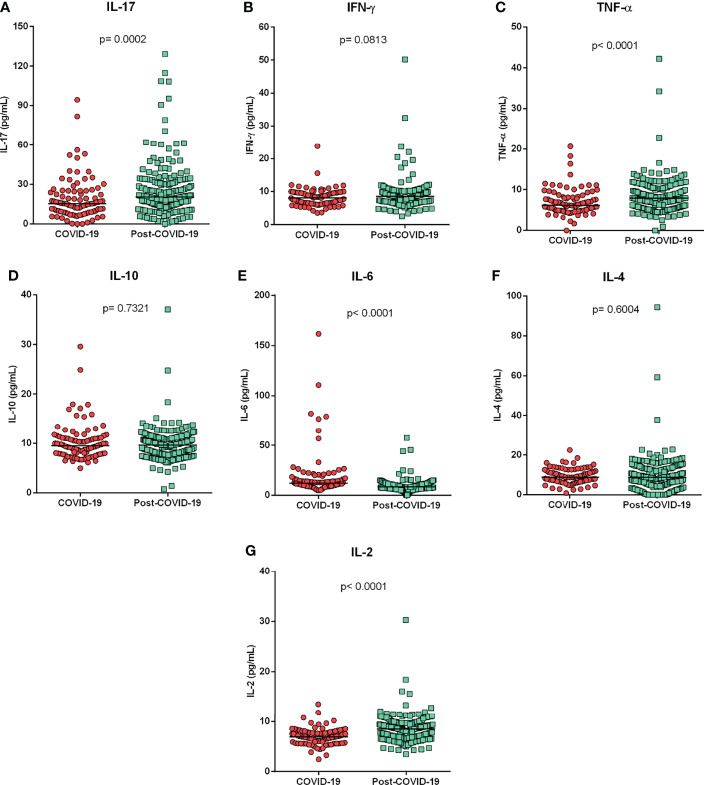
Cytokine levels according to different clinical manifestations of COVID-19. Comparison of cytokine levels **(A)** IL-17, **(B)** IFN-y, **(C)** TNF-a, **(D)** IL-10, **(E)** IL-6, **(F)** IL-4 and **(G)** IL-2, between patients with acute COVID-19 and individuals in the post-COVID-19 period. Mann-Whitney test.

Assessment of cytokine levels among patients with acute COVID-19 and individuals in the post-COVID-19 period showed significantly higher levels of IL-17 (p=0.0002; **Figure 1A**), TNF-α (p<0.0001; **Figure 1C**) and IL-2 (p<0.0001; **Figure 1G**) in the post-COVID-19 group and higher levels of IL-6 (p<0.0001; **Figure 1E**) in the acute COVID-19 group. The cytokines levels IFN-y (**Figure 1B**), IL-10 (**Figure 1D**) and IL-4 (**Figure 1F**) did not show significant differences between the groups.

**Figure 2:**
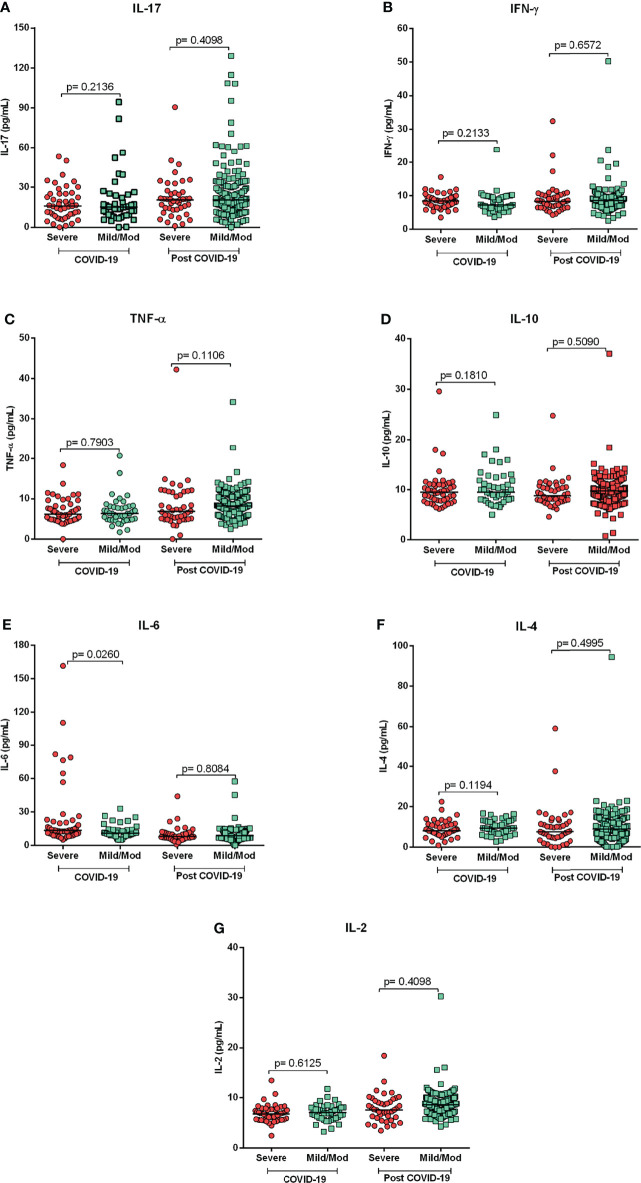
Cytokine profiles in acute and post-COVID-19 syndrome. Comparison of cytokine levels of patients with severe and mild/moderate COVID-19 and post- COVID-19. Mild/Mod: mild/moderate. Mann-Whitney Test. **(A)** IL-17, **(B)** IFN-y, **(C)** TNF-a, **(D)** IL-10, **(E)** IL-6, **(F)** IL-4 and **(G)** IL-2.

Comparison of cytokine levels showed that patients with severe acute COVID-19 had significantly higher levels of IL-6 (p=0.0260; **Figure 2E**) as compared to mild/moderate group. Conversely, in the group of post-COVID-19 subjects, no significant differences in cytokine level among those with severe clinical forms compared to mild/moderate forms were observed (**Figures 2A–G**).

Some variables, such as sex, age and comorbidities (diabetes mellitus, hypertension, chronic kidney disease, obesity and immunosuppression), which can influence the immune response, were evaluated in relation to the clinical manifestations of patients with COVID-19 and levels of cytokines. When comparing the frequencies of the variables between the groups of patients with COVID-19, all variables showed significant differences (**Table 2**). The severe group had a higher frequency of males (p=0.0027), patients aged over 60 years (p<0.0001) and comorbidities (p<0.0001).

**Table 2:** Distribution of frequencies of epidemiological variables between groups with different clinical conditions of COVID-19.

Variables	Severe N = 91N (%)	Mild/Moderate N = 226N (%)	p
**Sex**			
Female	40 (43.96)	131 (58.00)	0.0027*
Male	51 (56.04)	95 (42.00)	
**Age** (years)			
21-40	22 (24.18)	106 (46.90)	<0.0001**
41-60	45 (49.45)	105 (46.46)	
>60	24 (26.37)	15 (6.64)	
**Comorbidities**			
Yes	49 (53.85)	61 (26.99)	<0.0001*
No	42 (46.15)	165 (73.01)	

N, number of individuals/samples; * Fisher’s exact test; ** Chi-square test.

**Figure 3:**
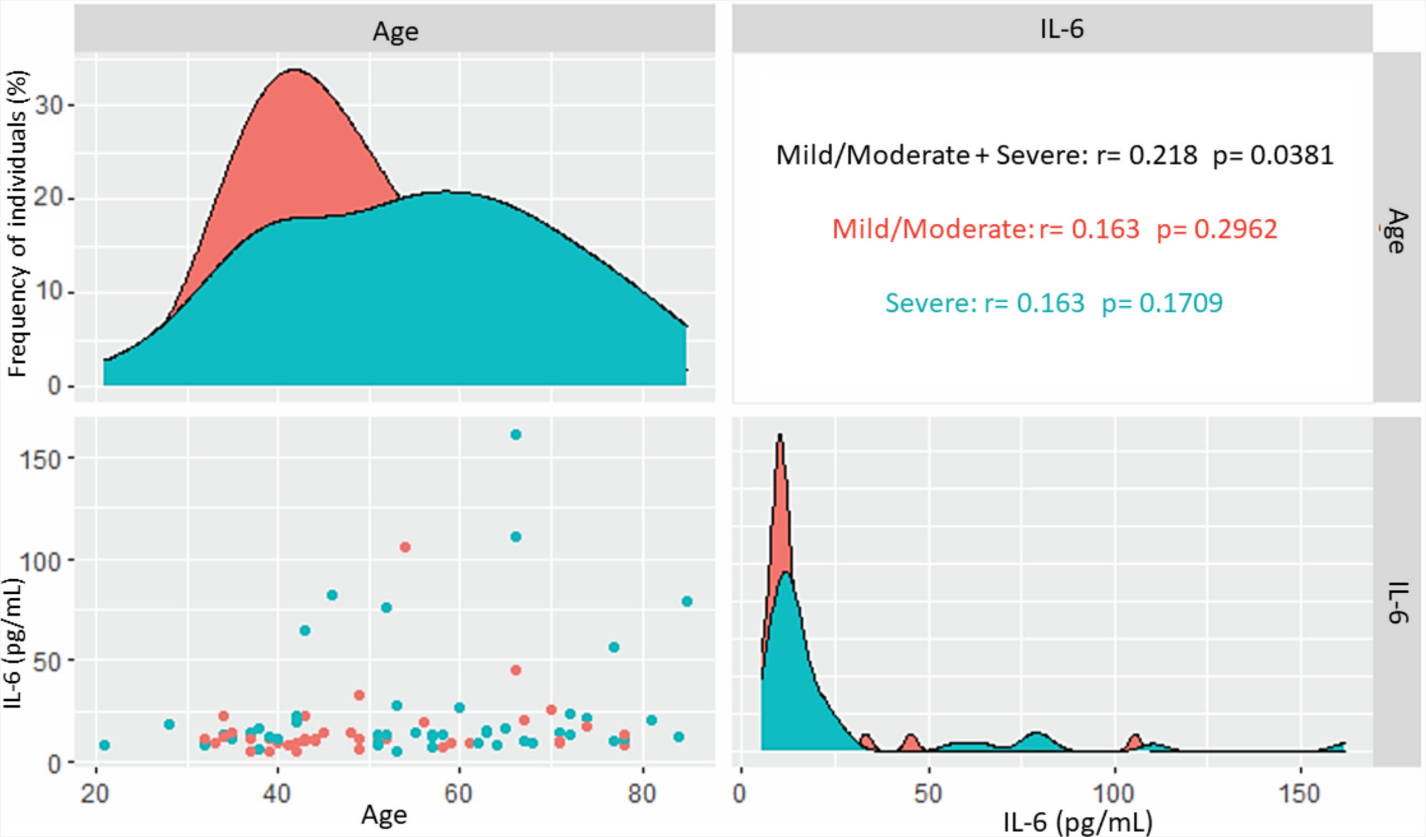
Correlation of IL-6 levels and age in acute COVID-19. Correlation of all patients and patients with mild/moderate and severe forms of the disease.

In the group with acute COVID-19, cytokine levels were evaluated in relation to epidemiological variables (**Table 3**). Patients aged 21-40 years had higher levels of TNF-α (p= 0.0257), and those over age 60 had higher levels of IL-6, with a p value close to statistical significance (p=0.0636). Patients without comorbidities had higher levels of TNF-α (p=0.0030), IL-4 (p=0.0020) and IL-2 (p=0.0021); IL-6 levels were higher in patients with comorbidities, but without statistical significance (p=0.0891). Analysis of IL-6 levels with the age in those with acute COVID-19 showed a slightly positive correlation (r= 0.218; p= 0.0381), but there was no correlation between the variables when the evaluation was performed according to the different clinical forms of the disease (**Figure 3**). IL-6 levels were higher in hospitalized patients admitted to the ICU (median= 15.41) than in those who did not require intensive care (median= 12.59), but the difference was not significant (p= 0.3353).

**Table 3:** Comparison of cytokine levels in relation to epidemiological variables among patients with acute COVID-19.

Variables	IL-17 (pg/mL) Median (IIQ)	IFN-γ (pg/mL) Median (IIQ)	TNF-α (pg/mL) Median (IIQ)	IL-10 (pg/mL) Median (IIQ)	IL-6 (pg/mL) Median (IIQ)	IL-4 (pg/mL) Median (IIQ)	IL-2 (pg/mL) Median (IIQ)
Sex*							
Female	15.44 (17.66)	8.21 (3.71)	6.00 (3.50)	8.96 (3.82)	12.56 (8.84)	9.43 (7.02)	6.76 (2.03)
Male	15.45 (11.58)	8.24 (3.67)	6.25 (3.28)	9.95 (3.25)	11.70 (7.90)	8.09 (4.75)	6.96 (1.80)
p	0.9735	0.7270	0.7688	0.1618	0.8733	0.2121	0.3360
**Age** (years)**							
21-40	15.80 (9.30)	8.37 (3.09)	7.67 (3.94)	8.56 (2.32)	11.39 (4.27)	10.34 (4.82)	7.44 (2.10)
41-60	15.31 (16.76)	8.35 (3.29)	6.78 (2.94)	9.59 (3.50)	12.71 (9.24)	8.46 (5.26)	6.88 (1.95)
>60	11.74 (16.76)	8.05 (3.05)	5.95 (3.14)	10.43 (2.80)	14.51 (11.97)	7.86 (5.17)	5.52 (1.77)
p	0.6310	0.9853	0.0257	0.1674	0.0636	0.2700	0.1263
**Comorbidities** *							
Yes	16.40 (17.03)	7.70 (2.87)	5.69 (2.33)	9.63 (4.67)	13.13 (11.68)	7.27 (4.88)	6.61 (1.73)
No	15.45 (9.30)	8.65 (3.02)	7.05 (3.59)	9.39 (3.08)	11.35 (4.96)	10.19 (4.84)	7.49 (1.68)
p	0.9922	0.2527	0.0030	0.9893	0.0891	0.0020	0.0021

IIQ, interquartile range; * Mann–Whitney test; ** Kruskal–Wallis test.

**Figure 4:**
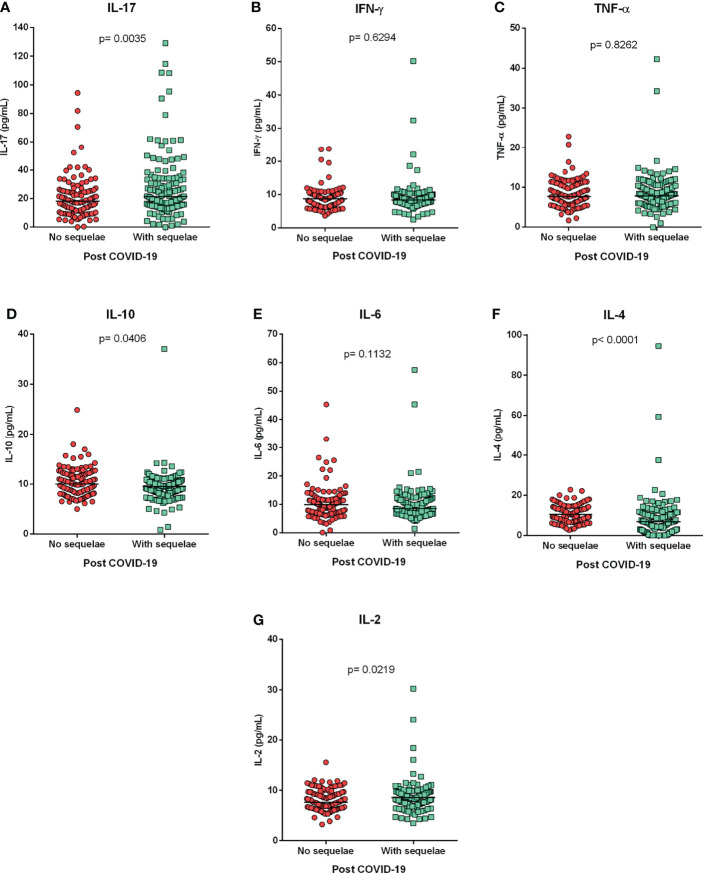
Cytokine profile in the long COVID-19. Comparison of cytokine levels **(A)** IL-17, **(B)** IFN-y, **(C)** TNF-a, **(D)** IL-10, **(E)** IL-6, **(F)** IL-4 and **(G)** IL-2, between individuals with and without post-COVID-19 symptoms (sequelae). Mann-Whitney test.

**Figure 5:**
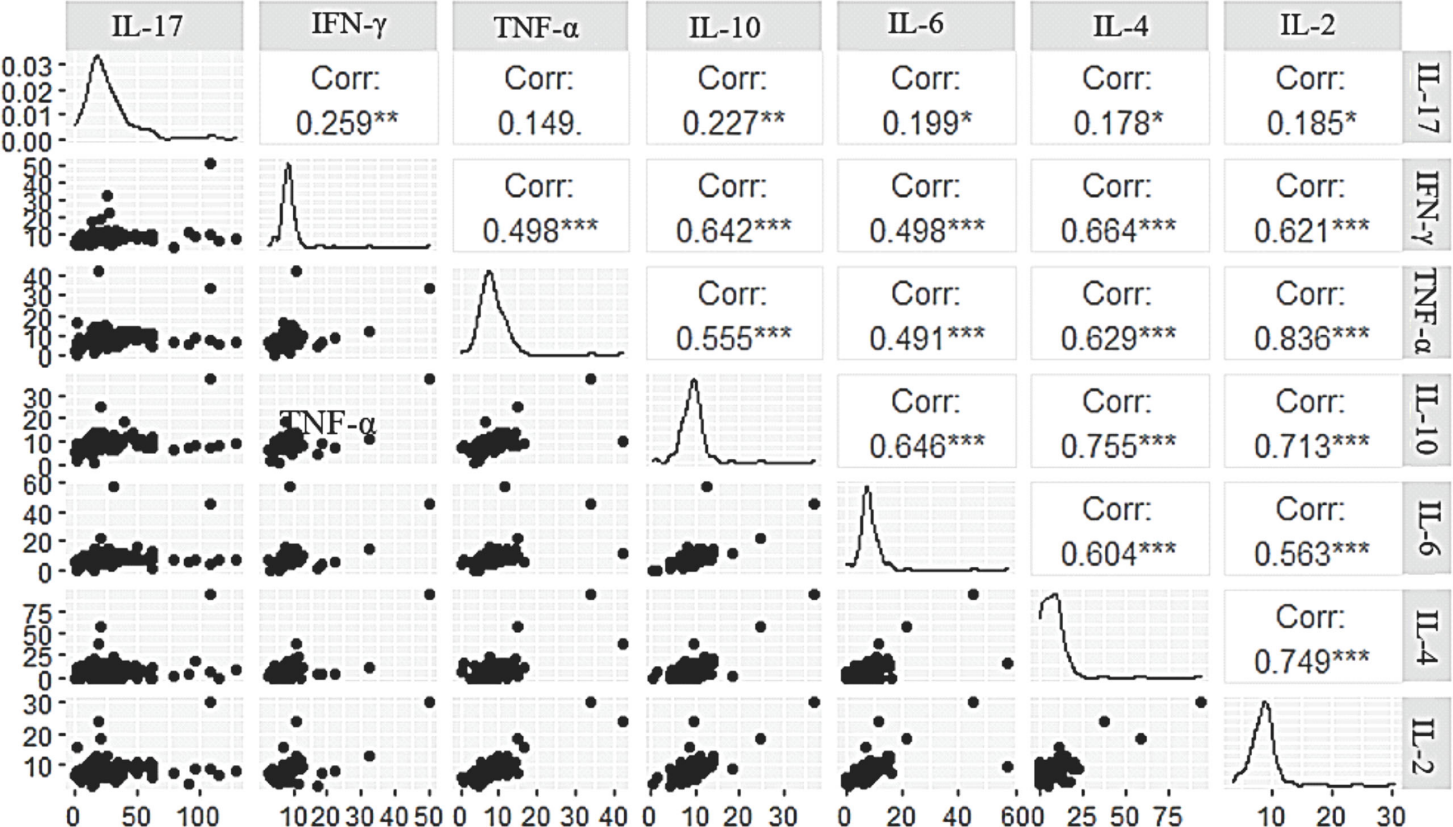
Correlogram of cytokine levels evaluated in long COVID-19. *p<0.01; **p<0.001 and ***p<0.0001

The post-COVID-19 group was divided into individuals who were undergoing medical follow-up for sequelae (long COVID) and those who had recovered and had no sequelae. Assessment of cytokine levels between these groups showed that individuals with long COVID (sequelae) had higher levels of IL-17 (p=0.0035; **Figure 4A**) and IL-2 (p=0.0219; **Figure 4G**) and that individuals without sequelae had higher levels of IL-10 (p=0.0406; **Figure 4D**) and IL-4 (p<0.0001; **Figure 4F**). A correlogram of cytokine levels in long COVID-19 patients revealed a positive correlation for most cytokines (**Figure 5**). The cytokines levels IFN-y (**Figure 4B**), TNF-a (**Figure 4C**) and IL-6 (**Figure 4E**) did not show significant differences between the groups.

## Discussion

The main risk factors for severe and long COVID-19 include age, male sex, smoking, presence of comorbidities (obesity, diabetes, hypertension, heart disease) and variations in the immune response of the host (Zhou et al., 2020; Ejaz et al., 2020; Wang et al., 2021; Chang et al., 2021). Evidence suggests that age is the most significant risk factor for the severe form of COVID-19 and related complications (Chen et al., 2021). In the present study, subjects in the post-COVID-19 group had higher levels of IL-17, TNF-α and IL-2 as compared to the acute COVID-19 group. In contrast, patients in the acute COVID-19 group had higher levels of IL-6. Younger patients had higher levels of TNF-α, and patients without comorbidities had higher levels of TNF-α, IL-4 and IL-2. Patients over age 60, with comorbidities, had higher levels of IL-6. In the post-COVID-19 group, subjects with long COVID-19 had higher levels of IL-17 and IL-2 and subjects without sequelae had higher levels of IL-10, IL-6 and IL- 4.

The death rate from COVID-19 is directly related to age, whereby older age is associated with greater risk. The rate varies from 0.4 to 3.3% in patients aged 40 years or younger, from 1.3 to 4.85% in those aged 40-50 years, 3.6-6.4% in those aged 60-70, 8.0 to 12.6% in those aged 70-80, and 14.8%-25.9% in those older than 80 (Chen et al., 2021). Overall, rates of hospitalizations, ICU admissions, and death from COVID-19 increase with age (CDC COVID-19 Response Team, 2020), which occurs because of immune system remodeling or immunosenescence, rendering individuals more susceptible to infections, with more severe disease symptoms and lower response to vaccination (Pawelec, 2018; Bichara et al., 2021a; Bichara et al., 2021b). IL-6 levels showed a slight correlation with advanced age in acute COVID-19. However, there was no correlation of cytokine level with age according to the different clinical forms, which suggests that age may contribute to the increase in IL-6 levels but that these two factors are not sufficient to define the severity of the disease. Although IL-6 levels were higher in older patients with acute COVID-19, cytokine levels showed no statistically significant difference between age groups in this group, possibly due to the small sample size, which is a limitation of the study.

In our study, patients with the severe form of acute COVID-19 had higher levels of IL-6 and had some type of comorbidity (diabetes mellitus, hypertension, obesity, and immunosuppression). In the evaluation of patients with acute COVID-19, regardless of disease severity, individuals with comorbidities had higher levels of IL-6 (but without statistical significance). Comorbidities can contribute to the development of COVID-19 and are associated with worse prognosis due to the establishment of complex inflammatory symptoms (Ejaz et al., 2020; Fang et al., 2020).

The IL-6 contributes to inflammation in all insulin target tissues, including fat, the liver, and muscle, indicating the role of IL-6 in driving obesity and in the pathogenesis of systemic insulin resistance (Rocha and Folco, 2011; El-Kadre and Tinoco, 2013). In addition to IL-6, variations in the levels of different cytokines are involved in the development of different comorbidities (Mirhafez et al., 2014; Donath et al., 2019; Moghbeli et al., 2021). Although the cytokines TNF-α, IL-4 and IL-2 were not associated with severe COVID-19 in the present study, lower levels of these cytokines were observed in patients with comorbidities who had acute COVID-19.

The main risk factors associated with COVID-19, related to advanced age and the presence of comorbidities, are conditions characterized by constant low-grade activation of the inflammatory response (Geerlings and Hoepelman, 1999; Maggio et al., 2006; Franceschi and Campisi, 2014; Rodríguez-Iturbe et al., 2014), which promote impairment of the general immune response to infection. This suggests that although the clinical profile of severe COVID-19 is induced by an increase in cytokine production, it may be that this increase occurs in a moderate way and is sufficient to intensify inflammatory processes already existing in patients with the aforementioned risk factors and deregulate homeostasis. Furthermore, corticosteroid use in critically ill patients may also be associated with lower levels of cytokines compared to those with less aggressive forms of the disease.

IL-6 also contributes to increase vascular permeability and cause interstitial edema and tissue damage (Marin et al., 2001; Riedemann et al., 2003; Lowe et al., 2004). An increase in these events is related to the severity of COVID-19 and is responsible for many deaths (Klok et al., 2020; Helms et al., 2020; Wool and Miller, 2021).

Although the severity of COVID-19 is related to risk factors such as advanced age and comorbidities, Del Valle et al. (2020) noted that elevated serum levels of IL-6, IL-8, and TNF-α in COVID-19 patients at the time of hospitalization were strong and independent predictors of patient survival. IL-6 was one of the most robust prognostic markers of survival (surpassing CRP, D-dimer and ferritin), and elevated IL-6 levels were associated with severity and predictive of poor outcome. In our study, among the cytokines evaluated, only high levels of IL-6 were associated with the severity of COVID-19, confirming the relevance of this cytokine in the outcome of the disease. In this sense, maintenance of high levels of IL-6 in patients with the severe form of COVID-19 might be mainly related to the consequence of the inflammatory process resulting from the main risk factors for the disease, the presence of different comorbidities and advanced age and will contribute to intensifying the inflammatory process and the development of severe clotting events.

Some studies have evaluated symptoms related to long COVID-19 (Fernández-de-Las-Peñas et al., 2021; Abdallah et al., 2021; Huang et al., 2021a; Huang et al., 2021b; Logue et al., 2021; Augustin et al., 2021; Shuwa et al., 2021). However, most of these studies evaluated the long COVID-19 symptoms among patients who were hospitalized, i.e., only from patients who had severe COVID-19. Augustin et al. (2021) evaluated post-COVID syndrome in outpatients, but in order to correlate clinical symptoms with IgG antibodies, they did not evaluate molecular markers of inflammation. Cytokine IL-6 has been proposed as a potential mediator of neuropsychiatric symptoms of long COVID-19, possibly related to its persistence (Kappelmann et al., 2021). This relationship was not observed in our study, since individuals in the post-COVID-19 group had lower levels of IL-6, compared to patients with acute COVID-19. Furthermore, in the post-COVID group there was no significant difference in IL-6 levels between individuals with and without sequelae in the COVID-19 group. The relationship of IL-6 levels with the development of symptoms in long COVID-10, including neuropsychiatric symptoms, needs to be better investigated.

Shuwa et al. (2021) identified a likely risk of developing prolonged symptoms of COVID-19 in hospitalized patients, noting that convalescent patients had a lower frequency of IL-10^+^ CD4^+^ T cells, IL-17^+^ CD4^+^ T cells, and IL-6^+^ B cells, compared to individuals with acute COVID-19. Although in our study we identified lower levels of IL-10 in the long COVID-19 group compared to subjects without sequelae in the post-COVID-19 group, IL-17 levels were higher in those subjects with long COVID-19. Furthermore, no difference was observed in IL-6 levels between the groups. The difference between the results presented here and those observed by Shuwa et al. (2021) may be due to the clinical moment at which the patients were analyzed. In our study, evaluated individuals in the post-COVID-19 period, including individuals with and without sequelae, as well as individuals who had acute COVID-19 with different clinical manifestations (mild, moderate and severe), showing that the development of long COVID-19 may not be related only with the severity of acute COVID-19. In addition, it was possible to identify immuno-inflammatory molecular patterns, through the characterization of the cytokine profile, which may be related to long COVID-19.

In relation to individuals in the post-COVID-19 group, higher levels of IL-10 and IL-4 were observed in the group that did not present sequelae after the disease. This suggests better control of the inflammatory process due to increased levels of anti-inflammatory cytokines in these individuals. Furthermore, individuals who continued to experience sequelae after infection (long COVID) had significantly higher levels of IL-17 and IL-2 and lower levels of Il-4 and IL-10, suggesting a possible “molecular signature” for long COVID characterized by a Th17 inflammatory profile with a reduced anti-inflammatory response mediated by IL-4 and IL-10, that must be confirmed by other studies enrolling a largest sample size.

The correlation of cytokine levels in the long COVID-19 group was positive among most of the cytokines evaluated. Hence, although the disease is associated with variations in levels of certain cytokines, it appears to induce an increase in levels of cytokines related to different types of inflammatory response. Post-COVID syndrome (long COVID) has been associated with residual inflammation, organ damage, preexisting health conditions or nonspecific effects due to hospitalization or prolonged ventilation (Moreno-Pérez et al., 2021), conditions easily related to the severe form of the disease. Nonetheless, it is important to emphasize that SARS-CoV-2 shows tropism for the nervous system, and neurological manifestations can be observed in patients with different clinical forms of the disease (mild, moderate and severe), ranging from anosmia, ageusia, headache, stroke, Guillain–Barré syndrome, seizure and encephalopathy (Camargo-Martínez et al., 2021). Thus, it is possible that microvascular inflammation in cells of the nervous system during infection may trigger mild symptoms of the disease (Lechner-Scott et al., 2021), which may persist even after the infection has resolved.

The consequences of the long COVID-19 are impacting health care resources, as up to 30% of the associated health burden may be due to prolonged disability induced by COVID-19 rather than mortality (Parums, 2021). Therefore, understanding the biological basis of long COVID-19 will allow us to identify factors that predispose to the development of long-term complications and guide effective therapies. In this sense, several studies have been developed, such as the REACT-GE study (in partnership with Genomics England) that aims to look for biological signatures that may be linked to the development of long COVID-19 and whether genes affect the severity of the COVID-19 and its long-term progression (Post-COVID-19 syndrome: in it for the long haul, 2021)

Some studies have evaluated the molecular signature of acute COVID-19 cytokines by different methodologies (Bergamaschi et al., 2021; Freire et al., 2021; Szabo et al., 2021; Schimke et al., 2022). In our study, IL-6 levels were associated with the severe form of acute COVID-19. Bergamaschi et al. (2021) also identified IL-6 persistence in severe disease, along with the TNF-α. In contrast, other studies have shown that disease severity was related to elevated levels of myeloid chemoattractants and neutrophil activation (Freire et al., 2021; Szabo et al., 2021; Schimke et al., 2022). Possible differences in results (molecular profiles) observed between different studies may be due to differences in methods used for investigation, different times of sample collection during infection, or even due to differences in the immunogenetic background of the investigated population. Therefore, identification of a molecular profile in relation to long COVID-19 still needs to be further investigated. Even though several studies have evaluated the main symptoms of long COVID-19 (Abdallah et al., 2021; Augustin et al., 2021; Darley et al., 2021; Fernández-de-Las-Peñas et al., 2021; Logue et al., 2021; Wu et al., 2021; Huang et al., 2021a; Huang et al., 2021b), none of them investigated the association of clinical manifestations with the immunoinflammatory profile of cytokines. Our study showed, for the first time, evidence for the existence of a long COVID-19-associated cytokine profile in a cohort of the Brazilian Amazon region. Further evaluated in studies conducted with larger cohorts from distinct geographic areas is needed.

Our results are important because it was possible to identify differences in cytokine synthesis and identify characteristic profiles in acute COVID-19 (higher levels of IL-6) and long COVID-19 (high levels of IL-2 and IL- 17).

In conclusion, the present study shows that advanced age, the presence of comorbidities and elevated serum IL-6 levels are associated with the severity of COVID-19 and represent good markers to differentiate severe COVID-19 from mild clinical forms. Furthermore, high serum levels of IL-17 and IL-2, as well as low levels of IL-4 and IL-10, appear to constitute a long COVID-19 cytokine profile (molecular signature), and identification of these markers as a potential target may establish more adequate treatment and prevention strategies for specific groups.

## Data Availability Statement

The original contributions presented in the study are included in the article/supplementary material. Further inquiries can be directed to the corresponding author.

## Ethics Statement

The studies involving human participants were reviewed and approved by National Research Ethics Committee (CAEE: 33470020.1001.0018). The patients/participants provided their written informed consent to participate in this study.

## Author Contributions

AVa, LF, IV and ES conceived of the project. AQ, AVa, IB-C, JQ and ES wrote and reviewed the manuscript. SL and MQ performed the statistical analyses. AS, JL, MT, MB, CB, MC, IB-C, JN, ES, ML, FC, MV, FR, KS, RS, CB, AB, AVe, MC, DV, CO, GV, FC, VP, PN, and IB-C collected the biological samples and performed the laboratory analyses. All authors reviewed and approved the article.

## Funding

The study was supported by the National Council for Scientific and Technological Development (CNPQ #401235/2020-3); Fundação Amazônia de Amparo a Estudos e Pesquisa do Pará (FAPESPA #005/2020 and #006/2020) and Secretaria de Estado de Ciência, Tecnologia e Educação Profissional e Tecnológica (#09/2021).

## Conflict of Interest

The authors declare that the research was conducted in the absence of any commercial or financial relationships that could be construed as a potential conflict of interest.

## Publisher’s Note

All claims expressed in this article are solely those of the authors and do not necessarily represent those of their affiliated organizations, or those of the publisher, the editors and the reviewers. Any product that may be evaluated in this article, or claim that may be made by its manufacturer, is not guaranteed or endorsed by the publisher.

## References

[B1] Post-COVID-19 syndrome: in it for the long haul. (2021). EBioMedicine 67, 103424. doi: 10.1016/j.ebiom.2021.103424 34051888 PMC8153191

[B2] Abdallah S. J. Voduc N. Corrales-Medina V. F. McGuinty M. Pratt A. Chopra A.. (2021). Symptoms, Pulmonary Function, and Functional Capacity Four Months After COVID-19. Ann. Am. Thorac. Soc 18, 1912–1917. doi: 10.1513/AnnalsATS.202012-1489RL 33872135 PMC8641826

[B3] Augustin M. Schommers P. Stecher M. Dewald F. Gieselmann L. Gruell H.. (2021). Post-COVID Syndrome in non-Hospitalised Patients With COVID-19: A Longitudinal Prospective Cohort Study. Lancet Reg. Health Eur. 6, 100122. doi: 10.1016/j.lanepe.2021.100122 34027514 PMC8129613

[B4] Bergamaschi L. Mescia F. Turner L. Hanson A. L. Kotagiri P. Dunmore B. J.. (2021). Longitudinal Analysis Reveals That Delayed Bystander CD8+ T Cell Activation and Early Immune Pathology Distinguish Severe COVID-19 From Mild Disease. Immunity 54, 1257–1275. doi: 10.1016/j.immuni.2021.05.010 34051148 PMC8125900

[B5] Bichara C. D. A. da Silva Graça Amoras E. Vaz G. L. da Silva Torres M. K. Queiroz M. A. F. do Amaral I. P. C.. (2021b). Dynamics of Anti-SARS-CoV-2 IgG Antibodies Post-COVID-19 in a Brazilian Amazon Population. BMC Infect. Dis. 21, 443. doi: 10.1186/s12879-021-06156-x 33992073 PMC8122196

[B6] Bichara C. D. A. Queiroz M. A. F. da Silva Graça Amoras E. Vaz G. L. Vallinoto I. M. V. C. Bichara C. N. C.. (2021a). Assessment of Anti-SARS-CoV-2 Antibodies Post-Coronavac Vaccination in the Amazon Region of Brazil. Vaccines (Basel) 9, 1169. doi: 10.3390/vaccines9101169 34696277 PMC8539673

[B7] Buszko M. Park J. H. Verthelyi D. Sen R. Young H. A. Rosenberg A. S. (2020). The Dynamic Changes in Cytokine Responses in COVID-19: A Snapshot of the Current State of Knowledge. Nat. Immunol. 21, 1146–1151. doi: 10.1038/s41590-020-0779-1 32855555

[B8] Camargo-Martínez W. Lozada-Martínez I. Escobar-Collazos A. Navarro-Coronado A. Moscote-Salazar L. Pacheco-Hernández A.. (2021). Post-COVID 19 Neurological Syndrome: Implications for Sequelae's Treatment. J. Clin. Neurosci. 88, 219–225. doi: 10.1016/j.jocn.2021.04.001 33992187 PMC8031003

[B9] CDC COVID-19 Response Team. (2020). Severe Outcomes Among Patients With Coronavirus Disease 2019 (COVID-19) - United States, February 12-March 16, 2020. MMWR Morb. Mortal. Wkly. Rep. 69, 343–346. doi: 10.15585/mmwr.mm6912e2 32214079 PMC7725513

[B10] Chang S. H. Minn D. Kim S. W. Kim Y. K. (2021). Inflammatory Markers and Cytokines in Moderate and Critical Cases of COVID-19. Clin. Lab. 67 (9). doi: 10.7754/Clin.Lab.2021.210142 34542974

[B11] Chen Y. Klein S. L. Garibaldi B. T. Li H. Wu C. Osevala N. M.. (2021). Aging in COVID-19: Vulnerability, Immunity and Intervention. Ageing Res. Rev. 65, 101205. doi: 10.1016/j.arr.2020.101205 33137510 PMC7604159

[B12] Collins F. S. (2021) NIH Launches New Initiative to Study “Long COVID” (Bethesda, MD, USA: National Institutes of Health). Available at: https://www.nih.gov/about-nih/who-we-are/nih-director/statements/nih-launches-new-initiative-study-long-covid (Accessed 27/05/2022).

[B13] Darley D. R. Dore G. J. Byrne A. L. Plit M. L. Brew B. J. Kelleher A.. (2021). Limited Recovery From Post-Acute Sequelae of SARS-CoV-2 at 8 Months in a Prospective Cohort. ERJ Open Res. 7, 00384–02021. doi: 10.1183/23120541.00384-2021 34725634 PMC8504133

[B14] Del Valle D. M. Kim-Schulze S. Huang H. H. Beckmann N. D. Nirenberg S. Wang B.. (2020). An Inflammatory Cytokine Signature Predicts COVID-19 Severity and Survival. Nat. Med. 6, 1636–1643. doi: 10.1038/s41591-020-1051-9 PMC7869028 32839624

[B15] Donath M. Y. Dinarello C. A. Mandrup-Poulsen T. (2019). Targeting Innate Immune Mediators in Type 1 and Type 2 Diabetes. Nat. Rev. Immunol. 19, 734–746. doi: 10.1038/s41577-019-0213-9 31501536

[B16] Ejaz H. Alsrhani A. Zafar A. Javed H. Junaid K. Abdalla A. E.. (2020). COVID-19 and Comorbidities: Deleterious Impact on Infected Patients. J. Infect. Public Health 13, 1833–1839. doi: 10.1016/j.jiph.2020.07.014 32788073 PMC7402107

[B17] El-Kadre L. J. Tinoco A. C. (2013). Interleukin-6 and Obesity: The Crosstalk Between Intestine, Pancreas and Liver. Curr. Opin. Clin. Nutr. Metab. Care 16, 564–568. doi: 10.1097/MCO.0b013e32836410e6 23924949

[B18] Fang X. Li S. Yu H. Wang P. Zhang Y. Chen Z.. (2020). Epidemiological, Comorbidity Factors With Severity and Prognosis of COVID-19: A Systematic Review and Meta-Analysis. Aging (Albany NY) 12, 12493–12503. doi: 10.18632/aging.103579 32658868 PMC7377860

[B19] Fernández-de-Las-Peñas C. Palacios-Ceña D. Gómez-Mayordomo V. Rodríuez-Jiménez J. Palacios-Ceña M. Velasco-Arribas M.. (2021). Long-Term Post-COVID Symptoms and Associated Risk Factors in Previously Hospitalized Patients: A Multicenter Study. J. Infect. 83, 237–279. doi: 10.1016/j.jinf.2021.04.036 PMC8110627 33984399

[B20] Franceschi C. Campisi J. (2014). Chronic Inflammation (Inflammaging) and its Potential Contribution to Age-Associated Diseases. J. Gerontol A Biol. Sci. Med. Sci. 69, S4–S9. doi: 10.1093/gerona/glu057 24833586

[B21] Freire P. P. Marques A. H. Baiocchi G. C. Schimke L. F. Fonseca D. L. Salgado R. C.. (2021). The Relationship Between Cytokine and Neutrophil Gene Network Distinguishes SARS-CoV-2-Infected Patients by Sex and Age. JCI Insight 6, e147535. doi: 10.1172/jci.insight.147535 PMC8262322 34027897

[B22] Geerlings S. E. Hoepelman A. I. (1999). Immune Dysfunction in Patients With Diabetes Mellitus (DM). FEMS Immunol. Med. Microbiol. 26, 259–265. doi: 10.1111/j.1574-695X.1999.tb01397.x 10575137

[B23] Helms J. Tacquard C. Severac F. Leonard-Lorant I. Ohana M. Delabranche X.. (2020). High Risk of Thrombosis in Patients With Severe SARS-CoV-2 Infection: A Multicenter Prospective Cohort Study. Intensive Care Med. 46, 1089–1098. doi: 10.1007/s00134-020-06062-x 32367170 PMC7197634

[B24] Huang C. Huang L. Wang Y. Li X. Ren L. Gu X.. (2021b). 6-Month Consequences of COVID-19 in Patients Discharged From Hospital: A Cohort Study. Lancet 397, 220–232. doi: 10.1016/S0140-6736(20)32656-8 33428867 PMC7833295

[B25] Huang C. Wang Y. Li X. Ren L. Zhao J. Hu Y.. (2021a). Clinical Features of Patients Infected With 2019 Novel Coronavirus in Wuhan, China. Lancet 395, 497–506. doi: 10.1016/S0140-6736(20)30183-5 PMC7159299 31986264

[B26] Kappelmann N. Dantzer R. Khandaker G. M. (2021). Interleukin-6 as Potential Mediator of Long-Term Neuropsychiatric Symptoms of COVID-19. Psychoneuroendocrinology 131, 105295. doi: 10.1016/j.psyneuen.2021.105295 34119855 PMC8172271

[B27] Kaur S. Bansal R. Kollimuttathuillam S. Gowda A. M. Singh B. Mehta D.. (2021). The Looming Storm: Blood and Cytokines in COVID-19. Blood Rev. 46, 100743. doi: 10.1016/j.blre.2020.100743 32829962 PMC7431319

[B28] Klok F. A. Kruip M. J. H. A. van der Meer N. J. M. Arbous M. S. Gommers D. A. M. P. J. Kant K. M.. (2020). Incidence of Thrombotic Complications in Critically Ill ICU Patients With COVID-19. Thromb. Res. 191, 145–147. doi: 10.1016/j.thromres.2020.04.013 32291094 PMC7146714

[B29] Kunnumakkara A. B. Rana V. Parama D. Banik K. Girisa S. Henamayee S.. (2021). COVID-19, Cytokines, Inflammation, and Spices: How are They Related? Life Sci. 284, 119201. doi: 10.1016/j.lfs.2021.119201 33607159 PMC7884924

[B30] Lechner-Scott J. Levy M. Hawkes C. Yeh A. Giovannoni G. (2021). Long COVID or Post COVID-19 Syndrome. Mult. Scler. Relat. Disord. 55, 103268. doi: 10.1016/j.msard.2021.103268 34601388 PMC8447548

[B31] Logue J. K. Franko N. M. McCulloch D. J. McDonald D. Magedson A. Wolf C. R.. (2021). Sequelae in Adults at 6 Months After COVID-19 Infection. JAMA Netw. Open 4, e210830. doi: 10.1001/jamanetworkopen.2021.0830 33606031 PMC7896197

[B32] Lowe G. D. Rumley A. McMahon A. D. Ford I. O'Reilly D. S. Packard C. J.. (2004). Interleukin-6, Fibrin D-Dimer, and Coagulation Factors VII and XIIa in Prediction of Coronary Heart Disease. Arterioscler. Thromb. Vasc. Biol. 24, 1529–1534. doi: 10.1161/01.ATV.0000135995.39488.6c 15205218

[B33] Maggio M. Guralnik J. M. Longo D. L. Ferrucci L. (2006). Interleukin-6 in Aging and Chronic Disease: A Magnificent Pathway. J. Gerontol A Biol. Sci. Med. Sci. 61, 575–584. doi: 10.1093/gerona/61.6.575 16799139 PMC2645627

[B34] Marin V. Montero-Julian F. A. Grès S. Boulay V. Bongrand P. Farnarier C.. (2001). The IL-6-Soluble IL-6Ralpha Autocrine Loop of Endothelial Activation as an Intermediate Between Acute and Chronic Inflammation: An Experimental Model Involving Thrombin. J. Immunol. 167, 3435–4342. doi: 10.4049/jimmunol.167.6.3435 11544336

[B35] Mirhafez S. R. Mohebati M. Feiz Disfani M. Saberi Karimian M. Ebrahimi M. Avan A.. (2014). An Imbalance in Serum Concentrations of Inflammatory and Anti-Inflammatory Cytokines in Hypertension. J. Am. Soc. Hypertens. 8, 614–623. doi: 10.1016/j.jash.2014.05.007 25224864

[B36] Moghbeli M. Khedmatgozar H. Yadegari M. Avan A. Ferns G. A. Ghayour Mobarhan M. (2021). Cytokines and the Immune Response in Obesity-Related Disorders. Adv. Clin. Chem. 101, 135–168. doi: 10.1016/bs.acc.2020.06.004 33706888

[B37] Moreno-Pérez O. Merino E. Leon-Ramirez J. M. Andres M. Ramos J. M. Arenas-Jiménez J.. (2021). Post-Acute COVID-19 Syndrome. Incidence and Risk Factors: A Mediterranean Cohort Study. J. Infect. 82, 378–383. doi: 10.1016/j.jinf.2021.01.004 33450302 PMC7802523

[B38] Mortaz E. Tabarsi P. Varahram M. Folkerts G. Adcock I. M. (2020). The Immune Response and Immunopathology of COVID-19. Front. Immunol. 11. doi: 10.3389/fimmu.2020.02037 PMC7479965 32983152

[B39] Nagy Á. Pongor S. Győrffy B. (2021). Different Mutations in SARS-CoV-2 Associate With Severe and Mild Outcome. Int. J. Antimicrob. Agents 57, 106272. doi: 10.1016/j.ijantimicag.2020 33347989 PMC7755579

[B40] Parums D. V. (2021). Editorial: Long COVID, or Post-COVID Syndrome, and the Global Impact on Health Care. Med. Sci. Monit. 27, e933446. doi: 10.12659/MSM.933446 34092779 PMC8194290

[B41] Pawelec G. (2018). Age and Immunity: What Is “Immunosenescence”? Exp. Gerontol. 105, 4–9. doi: 10.1016/j.exger.2017.10.024 29111233

[B42] Riedemann N. C. Neff T. A. Guo R. F. Bernacki K. D. Laudes I. J. Sarma J. V.. (2003). Protective Effects of IL-6 Blockade in Sepsis are Linked to Reduced C5a Receptor Expression. J. Immunol. 170, 503–507. doi: 10.4049/jimmunol.170.1.503 12496437

[B43] Rocha V. Z. Folco E. J. (2011). Inflammatory Concepts of Obesity. Int. J. Inflam. 2011, 529061. doi: 10.4061/2011/529061 21837268 PMC3151511

[B44] Rodríguez-Iturbe B. Pons H. Quiroz Y. Johnson R. J. (2014). The Immunological Basis of Hypertension. Am. J. Hypertens. 27, 1327–1337. doi: 10.1093/ajh/hpu142 25150828 PMC4263946

[B45] Schimke L. F. Marques A. H. C. Baiocchi G. C. de Souza Prado C. A. Fonseca D. L. M.. (2022). Severe COVID-19 Shares a Common Neutrophil Activation Signature With Other Acute Inflammatory States. Cells 11, 847. doi: 10.3390/cells11050847 35269470 PMC8909161

[B46] Shuwa H. A. Shaw T. N. Knight S. B. Wemyss K. McClure F. A. Pearmain L.. (2021). Alterations in T and B Cell Function Persist in Convalescent COVID-19 Patients. Med. (N Y) 2, 720–735.e4. doi: 10.1016/j.medj.2021.03.013 33821250 PMC8011689

[B47] Szabo P. A. Dogra P. Gray J. I. Wells S. B. Connors T. J. Weisberg S. P.. (2021). Longitudinal Profiling of Respiratory and Systemic Immune Responses Reveals Myeloid Cell-Driven Lung Inflammation in Severe COVID-19. Immunity 54, 797–814. doi: 10.1016/j.immuni.2021.03.005 33765436 PMC7951561

[B48] Teijaro J. R. (2017). Cytokine Storms in Infectious Diseases. Semin. Immunopathol. 39, 501–503. doi: 10.1007/s00281-017-0640-2 28674818 PMC7079934

[B49] Torres M. K. S. Bichara C. D. A. Almeida M. N. S. Vallinoto M. C. Queiroz M. A. F. Vallinoto I. M. V. C.. (2022). The Complexity of SARS-CoV-2 Infection and the COVID-19 Pandemic. Front. Microbiol. 13. doi: 10.3389/fmicb.2022.789882 PMC8870622 35222327

[B50] Wang F. Cao J. Yu Y. Ding J. Eshak E. S. Liu K.. (2020). Epidemiological Characteristics of Patients With Severe COVID-19 Infection in Wuhan, China: Evidence From a Retrospective Observational Study. Int. J. Epidemiol. 49, 1940–1950. doi: 10.1093/ije/dyaa180 PMC7665537 33150437

[B51] WHO (2021) WHO Global Clinical Platform for the Clinical Characterization of COVID-19: Statistical Analysis Plan. Available at: https://www.who.int/publications/i/item/WHO-2019-nCoV-Clinical-Analytic-plan-2021.1 (Accessed 15/02/2022).

[B52] WHO (2022) WHO Coronavirus (COVID-19) Dashboard. Available at: https://covid19.who.int/ (Accessed 08/02/2022).

[B53] Wool G. D. Miller J. L. (2021). The Impact of COVID-19 Disease on Platelets and Coagulation. Pathobiology 88, 15–27. doi: 10.1159/000512007 33049751 PMC7649697

[B54] Wu L. Wu Y. Xiong H. Mei B. You T. (2021). Persistence of Symptoms After Discharge of Patients Hospitalized Due to COVID-19. Front. Med. (Lausanne) 8. doi: 10.3389/fmed.2021.761314 PMC8645792 34881263

[B55] Zhou F. Yu T. Du R. Fan G. Liu Y. Liu Z.. (2020). Clinical Course and Risk Factors for Mortality of Adult Inpatients With COVID-19 in Wuhan, China: A Retrospective Cohort Study. Lancet 395, 1054–1062. doi: 10.1016/S0140-6736(20)30566-3 32171076 PMC7270627

